# A Novel Multi-Scale Ceramic-Based Array (SiCb+B_4_C_p_)/7075Al as Promising Materials for Armor Structure

**DOI:** 10.3390/ma16175796

**Published:** 2023-08-24

**Authors:** Tian Luo, Zhenlong Chao, Shanqi Du, Longtao Jiang, Shengpeng Chen, Runwei Zhang, Huimin Han, Bingzhuo Han, Zhiwei Wang, Guoqin Chen, Yong Mei

**Affiliations:** 1 School of Materials Science and Engineering, Harbin Institute of Technology, Harbin 150001, China; luotian1452@gmail.com (T.L.); 20b909008@stu.hit.edu.com (S.D.); zrw920127@163.com (R.Z.); hanhm1019@163.com (H.H.); 20b909002@stu.hit.edu.cn (B.H.); 18353591602@163.com (Z.W.); chenguoqin@hit.edu.cn (G.C.); 2 School of Astronautics, Harbin Institute of Technology, Harbin 150001, China; shengpeng.chen@hit.edu.cn; 3 State Key Laboratory of Advanced Welding and Joining, Harbin Institute of Technology, Harbin 150001, China; 4 No.52 Institute of China Ordnance Industries, Yantai 264003, China; 5 Institute of Defense Engineering, Academy of Military Science, People’s Liberation Army, Beijing 100036, China

**Keywords:** Al matrix composites, 12.7 mm API, ballistic performance, finite element simulation, LS-DYNA

## Abstract

Ceramic panel collapse will easily lead to the failure of traditional targets. One strategy to solve this problem is to use separate ceramic units as armor panels. Based on this idea, we propose an aluminum matrix composite using pressure infiltration, containing an array of ceramic balls, the reinforcement of which consists of centimeter-scale SiC balls and micron-scale B_4_C particles. Three different array layouts were designed and fabricated: compact balls in the front panel (F-C), non-compact balls in the front panel (F-NC), and compact balls inside the target (I-C). The penetration resistance properties were tested using a 12.7 mm armor-piercing incendiary (API). The results show that there are no significant internal defects, and the ceramic balls are well-bonded with the matrix composite. The F-NC structure behaves the best penetration resistance with minimal overall damage; the I-C structure has a large area of spalling and the most serious damage. Finite element simulation reveals that the ceramic balls play a major role in projectile erosion; in the non-compact structure, the composite materials between the ceramic balls can effectively disperse the stress, thereby avoiding the damage caused by direct contact between ceramic balls and improving the efficiency of ceramic ball erosion projectiles. Furthermore, it is essential to have a certain thickness of supporting materials to prevent spalling failure caused by stress wave transmission during penetration. This multi-scale composite exhibits excellent ballistic performance, providing valuable insights for developing anti-penetration composite armor in future applications.

## 1. Introduction

In the field of armor materials, with the advancement of anti-armor weapon development, it is difficult for a single material to meet the growing protection demands; thereby, composite samples prepared for being used in the fabrication of armor have received attention [1,2,3,4]. These composite samples, combining various materials, can fully exploit the advantageous properties of each material to achieve excellent protection performance while maintaining the integrity of the target plate. Currently, major types of composite armor include lattice armor [5,6], biomimetic armor [7,8], functionally graded armor [9,10], and ceramic composite armor [11,12,13].

The mainstream ceramic composite armor primarily consists of ceramic front panels and polymer rear materials [1,14,15]. Ceramic materials are widely applied in the armor domain due to their excellent properties, such as lightweight and high strength. However, their brittleness poses a challenge as localized cracks can accelerate the overall failure of the panel, often resulting in extensive collapse during penetration, which hinders multi-hit capability [12,16]. To address this issue, front panels have evolved from being solely composed of ceramic plates to include ordered array structures of ceramic tiles, balls, columns, and particles [17]. The utilization of independent ceramic units can effectively mitigate the failure of ceramic panels, thereby maximizing the erosion effect of the ceramic layer against projectiles. Additionally, this approach is beneficial for harnessing the potential of composite armor in countering multi-hit threats. In this regard, spherical shapes can effectively prevent stress concentration-induced damage caused by shape edges.

Similarly, the backing materials have diversified into various material systems, such as aluminum alloys and their composites, titanium alloys, and high-performance fiber materials [18]. Titanium alloys may exhibit sudden failure during penetration due to adiabatic shear instability [19,20]. Fiber composite materials have relatively lower shear strength and face challenges related to high production costs and limited environmental adaptability [4,21,22]. In contrast, on top of the high strength, low density, and low cost of aluminum alloy, aluminum composite materials offer excellent design flexibility, providing greater possibilities for optimizing the structure and performance of composite armor. Considering these factors, we chose independent ceramic ball units constrained by high-strength aluminum composites to obtain an ideal composite armor.

The penetration resistance mechanism of array structures has been widely studied [1,17,23]. Hu et al. [24] utilized squeeze casting infiltration to prepare a composite material consisting of 6 mm Al_2_O_3_ ceramic balls constrained within a 6061A1 matrix and subjected them to ballistic testing using a 12.7 mm incendiary projectile and a 30 mm armor-piercing projectile. The results revealed that the collisions between neighboring balls effectively dispersed the load brought by the projectile, and the alternate arrangement of the ductile alloy and the high-strength ceramic balls played a role in energy dispersion and accelerating the shockwave reflection, which improves the penetration resistance.

Based on the above anti-penetration mechanism, some studies have also optimized the size and shape of ceramic units [25,26,27,28]. Liu et al. [25] employed a combined experimental and simulation approach to investigate the influence of ceramic ball size on ballistic performance. It was found that reducing the size of ceramic balls within a certain range (6–20 mm) could enhance the penetration resistance of the target plate. Jiang et al. [26] discussed the projectile deflection caused by specially shaped ceramics, which in turn optimized the anti-penetration performance. However, current research in this field has predominantly emphasized the optimization of ceramic balls, with relatively limited investigations into the synergistic effects between ceramic ball arrays and constraint materials.

In order to investigate the synergistic effects between ceramic ball arrays and constraint materials during penetration, this study designed and fabricated composite targets with three different arrangements of ceramic balls, followed by ballistic testing. The numerical model was established using LS-DYNA to analyze the velocity-time history of the projectile, energy variations in different components, and damage patterns during the penetration process. Experimental and simulation results demonstrate that the ceramic balls’ arrangement can significantly influence composite armor’s ballistic resistance, which provides experimental and theoretical support for the structural optimization of the composite target.

## 2. Materials and Methods

### 2.1. Sample Design and Fabrication

**Figure 1:**
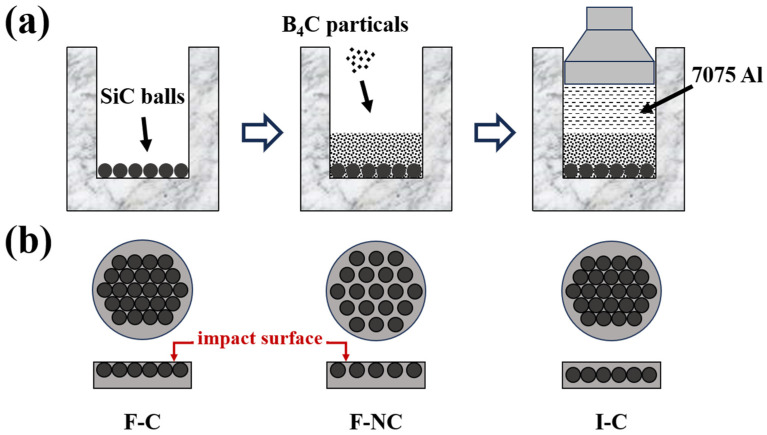
Schematic illustration for the composite armor. (**a**) Fabrication procedure, (**b**) Structure.

A target plate thickness of 20 mm was chosen to investigate the optimal arrangement of ceramic balls under the condition of equal areal density (~55 kg/m^2^). Figure 1 shows the preparation process and structural schematics of the designed composite armor. 8 mm SiC ceramic balls (Yangzhou Northern Sanshan Industrial Ceramics Co., Ltd., Yangzhou, China) were selected because of their high strength and mature sintering process. Additionally, the spherical shape can minimize the premature damage caused by the stress concentration within the ceramic during penetration. Due to its high strength and excellent penetration resistance, B_4_C (Mudanjiang Jingang Diamond Boron Carbide Co., Ltd., Mudanjiang, China) was chosen as the reinforcement in the composite, serving as both support and constraint. The B_4_C particle D50 size is 17.5 μm, and the volume fraction of B_4_C in the composite material is 50%.

For material preparation, SiC ceramic balls were first arranged inside the mold, and the 3D printing mold was used to achieve equal spacing of the balls (2 mm); then, an appropriate amount of B_4_C powder was placed inside the mold, followed by pouring molten 7075 aluminum (Northeast Light Alloy Co., Ltd., Harbin, China) into the mold. The target plate was then fabricated as an integrated structure via pressure infiltration. A more detailed preparation process was described in previous works [9,10]. The specimens were subjected to T6 heat treatment: solution treated at 475 °C for 1 h, after which water-quenched and aged at 120 °C for 24 h. The three different structures are labeled as F-C for compact balls in the front, F-NC for non-compact balls in the front, and I-C for compact balls inside the target. Table 1 gives the details of the produced composite armor.

### 2.2. Ballistic Impact Test

**Figure 2:**
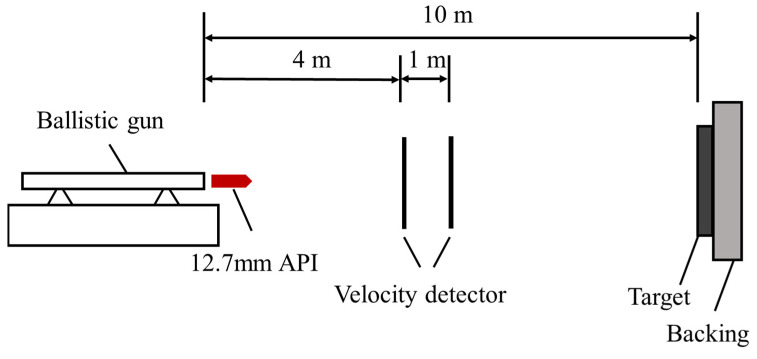
The schematic diagram of the ballistic impact test.

Figure 2 shows the schematic diagram of the ballistic test setup, including a ballistic gun, velocity measuring system, and target plate support. The round target plate (D = 130 mm) is supported by a thick steel plate. The 12.7 mm armor-piercing incendiary projectile (API) was fired toward the center of the armor sample at a velocity of 818 m/s, with a distance of 10 m between the ballistic gun and the target. Each experimental condition yielded at least two valid data.

The anti-penetration performance is evaluated using the dimension of the damaged area and the remaining depth of penetration (DOP) into the backing material. Due to the irregular shape of the damaged area, we need to find a more reasonable way to describe its dimensions. According to the reference [9], the diameter of the damaged area *D* was quantified using the entrance diameter (*D*_1_) and exit diameter (*D*_2_) of the projectile holes, which is given by:

(1) $$D=\sqrt{\left(D_{1}^{2}+D_{2}^{2}\right)/2}$$

*D*_1_ presents the entrance diameter (on the front plane), while *D*_2_ presents the exit diameter (on the rear plane) of the projectile hole in the target plate. The calculation formulas for *D*_1_ and *D*_2_ are as follows:

(2) $$D_{i}=\sqrt{\left(D_{x}^{2}+D_{y}^{2}\right)/2},i=1,2$$

**Figure 3:**
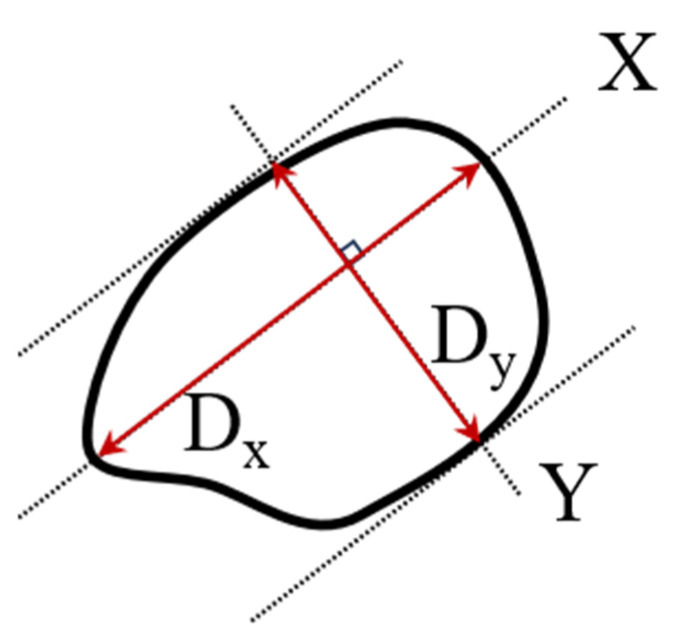
Schematic diagram of material damage diameter measurement.

*D_x_* denotes the longest distance within the damaged area, and *D_y_* represents the distance between the tangent lines parallel to the *X*-axis along the damaged area, as shown in Figure 3.

### 2.3. Numerical Simulation

#### 2.3.1. Model Specifications

**Figure 4:**
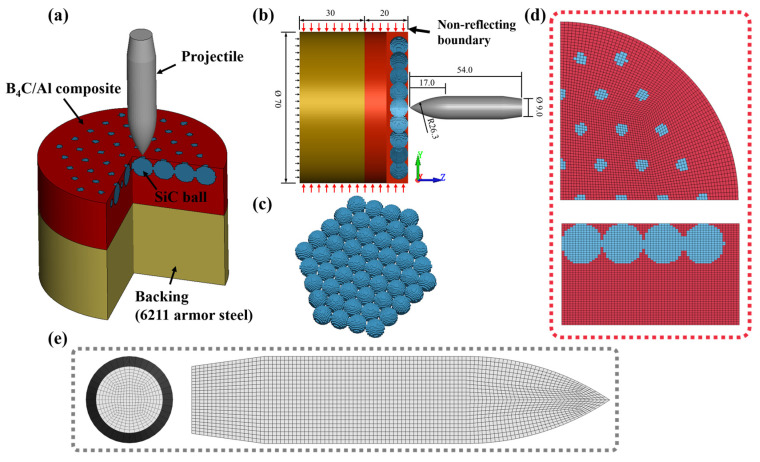
Typical finite element model. (**a**) Entire model, (**b**) Model size and boundary conditions, (**c**) SiC balls model, (**d**) Target plate mesh detail, (**e**) projectile mesh detail.

The numerical simulations were performed using LS-DYNA via dynamics explicit to understanding the anti-penetration mechanism of different structures against 12.7 mm API. It has been demonstrated that using the Lagrange finite element technique for the dynamic loading processes at high-speed events could provide reliable predictions. A three-dimensional (3D) finite element model was employed to build the projectile and targets, as shown in Figure 4, containing three parts: the bullet, the cylindrical target plate (ceramic balls and composite material), and the backing plate. Figure 4a,b show the detailed morphology, boundary conditions, and dimensions of the projectile–target structure. The displacement of the nodes at the bottom of the backing target plate is constrained in the z-direction, and a reflection-free boundary condition is imposed on the peripheral nodes of the cylindric target.

Figure 4 shows the finite element model of the projectile and target, which was meshed using a Lagrange eight-node solid element with full integration. The projectile–target structure was meshed uniformly in order to maintain numerical accuracy. To avoid mesh distortion, a pixel point ball was used to approximate it, as shown in Figure 4c. We select suitable element size dimensions of 0.5 × 0.5 × 0.35 mm based on the convergence study [17,22]. More detailed information about the mesh of the projectile–target structure is shown in Figure 4d,e.

#### 2.3.2. The Contact Types

The dynamic erosion contact (ERODING_SURFACE_TO_SURFACE_CONTACT) is applied to achieve the interaction between the projectile and target plate, as well as the components of the projectile–target structure itself. The contact strength of the ceramic balls and the composite material is described using commercial software LS-DYNA R12 keywords SURFACE_TO_SURFACE_TIEBREAK_CONTACT. The element deletion technique is introduced to simulate the potential crack initiation and propagation. When the damage of an element exceeds the critical value defined by keywords ADD_EROSION and the damage model included in the constitutive equation of the material in LS-DYNA, all the components of stress in that element are reset to 0, the material fails, and the element is deleted.

The ERODING_SURFACE_TO_SURFACE_CONTACT keyword is particularly useful for simulating complex contact behavior in multi-body systems, especially in cases involving material erosion, frictional wear, and surface damage [22,29,30]. In contrast, The SURFACE_TO_SURFACE_TIEBREAK_CONTACT keyword is used to deal with prioritization and competing conditions between multiple contact surfaces to ensure the accuracy and stability of simulations in complex contact situations. These prioritization rules can be determined based on a number of conditions, such as normal force, shear force, friction, etc. [31,32,33].

It must be pointed out that the element deletion technique is just a feasible approach to realize material separation, and it suffers from mass, momentum, energy loss, etc. To reduce the influence of element elimination on the impact, elements in the possible fracture region should be controlled to a sufficiently small size within an allowable computational expense. Due to the non-uniformity of the structure, there are many possibilities for the projectile impacting sites on the composite armor surface. To eliminate variances caused by the bullets hitting different positions, we made all the bullets hit the center of the ceramic ball directly at 818 m/s.

#### 2.3.3. Material Parameters

In the numerical model, SiC ceramic balls were modeled by the Johnson Holmquist Ceramics constitutive model (JH-2) [11], while the projectile, B_4_C/Al composite material, and the backing plate 6211 armor steel were modeled using the Johnson–Cook model (J–C) [9] that is able to characterize metal materials well undergoing large strains, high strain rates, and high temperatures. The parameters of the J–C and JH-2 models are listed in Table 2 and Table 3.

## 3. Results

### 3.1. Structure Characterization

**Figure 5:**
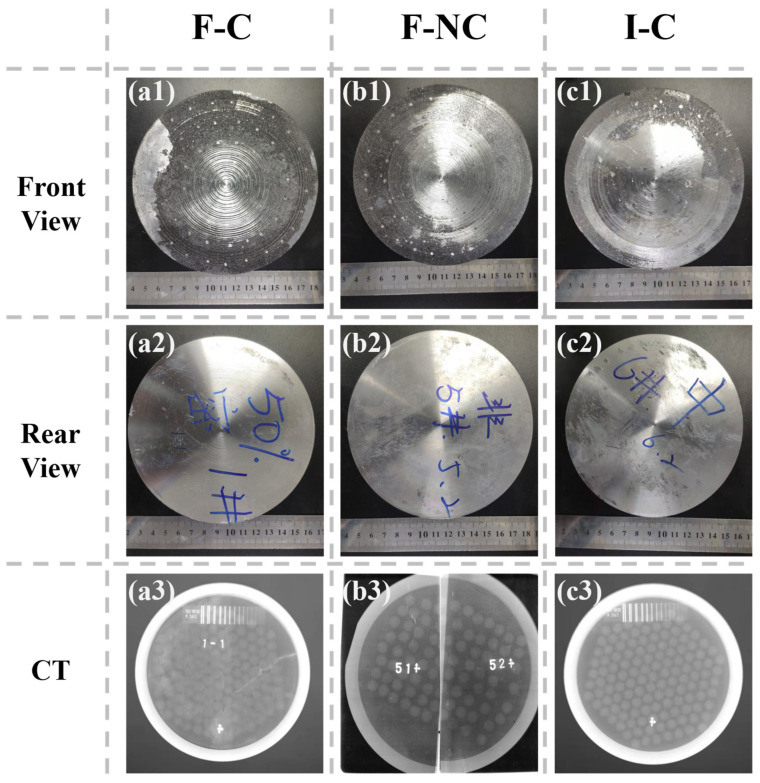
Front, rear view, and CT photos of composite armor. (**a1**–**a3**) F-C structure, (**b1**–**b3**) F-NC structure, (**c1**–**c3**) I-C structure.

Figure 5 presents the pictures of the composite armor before the ballistic test. The macroscopic surface of the target plate exhibits no discernible defects. Based on the CT images, it can be observed that in the F-C and I-C structures, the ceramic balls are compactly arranged as expected. Only in the F-NC structure does the position of ceramic balls vary slightly during the aluminum infiltration processes. The ceramic ball gaps are still filled with composite material in accordance with the expected design.

**Figure 6:**
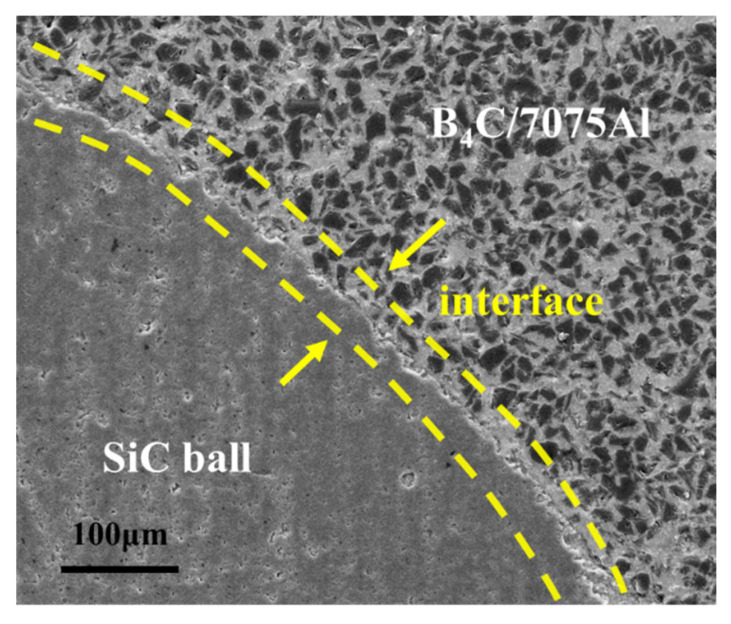
Interface between the SiC ball and B_4_C/7075Al composite.

Characterization of the target plate microstructure was also performed, and the results are shown in Figure 6. Within the composite material, B_4_C particles were uniformly distributed and exhibited good bonding with the 7075Al matrix without any significant voids or defects observed. Although there were a few pores present within the SiC ceramic balls, no noticeable cracks were detected. The intact structure of the ceramic balls helps prevent premature failure during the penetration process, thereby facilitating their resistance to penetration. Additionally, under the influence of pressure, the aluminum matrix and ceramic balls exhibited mutual infiltration, forming a tight metallurgical bond. Furthermore, surface undulations were observed on the ceramic balls, providing favorable conditions for mechanical interlocking between the composite material and the ceramic balls. These factors contribute to the effective support and constraint provided by the composite material to the ceramic balls.

Traditional composite armor, with polymer materials such as epoxy as the representative constraint material, exhibits disadvantages such as low strength and poor bonding with ceramic despite their low density. As a result, the penetration resistance performance of ceramics cannot be fully utilized [1,17,34]. In this study, the pressure infiltration process was employed to achieve an ideal interface bonding between the ceramic balls and the composite material. The high-strength composite material provides excellent support and constraint for the ceramic balls, thereby enhancing the projectile erosion effect of the ceramic balls and improving the overall ballistic performance.

### 3.2. Ballistic Performance

Ballistic testing was conducted on composite armor with different array arrangements. Due to experimental conditions, complete fragments were not collected from some target plates. In this study, the anti-penetration performance of the target plates was comprehensively evaluated based on the remaining depth of penetration (DOP) in the backing plate and the damage to the target plates. The specific penetration test results are presented in Table 4.

From the perspective of residual penetration depth in the backing plate, the F-NC target plate exhibited the smallest DOP, indicating minimal remaining energy of the projectile upon reaching the backing plate. Furthermore, no significant spallation occurred in the target plate, demonstrating excellent resistance to penetration.

**Figure 7:**
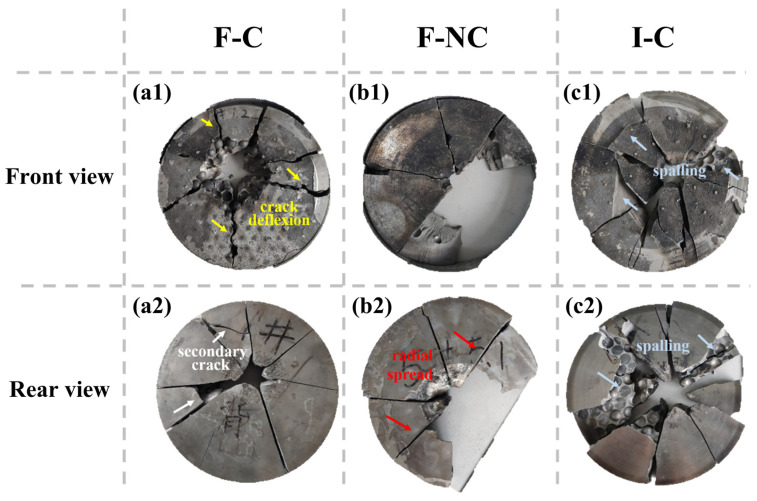
Front and rear view of armor after ballistic testing. (**a1**,**a2**) F-C structure, (**b1**,**b2**) F-NC structure, (**c1,c2**) I-C structure.

Regarding the damage condition shown in Figure 7, the F-C and F-NC target plates exhibited relatively fewer fragmented pieces and overall minor damage. In the F-C target plate, cracks propagated primarily radially outward from the impact point, with a few secondary cracks formed during crack expansion. Due to the rigid contact between the ceramic balls on the front plate, some ceramic balls collapsed near the impact point, resulting in spalling and the formation of enlarged holes. However, no spalling damage occurred on the rear surface of the target plate, indicating its effective load-bearing capability against the projectile’s impact.

Compared to the F-C target plate, the majority of ceramic balls near the impact point on the I-C target plate either detached from the matrix or were torn in half during the propagation of the stress wave. This results in extensive spalling on both the impact surface and the rear surface of the target plate, leading to a larger area of damage.

### 3.3. Numerical Simulation

#### 3.3.1. Validation of Material Parameters and Models

**Figure 8:**
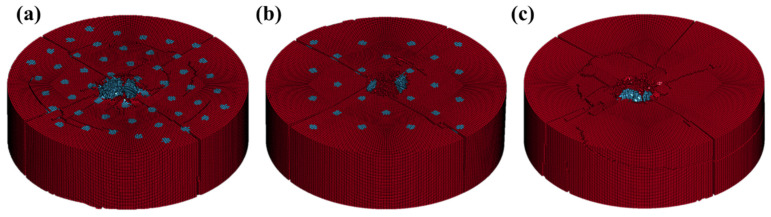
Damage of the target plates after penetration by FEM. (**a**) F-C structure, (**b**) F-NC structure, (**c**) I-C structure.

We validated the accuracy of material parameters and finite element models using the remaining depth of penetration (DOP) and macroscopic damage. In ballistic experiments, DOP is an important indicator, referring to the depth of penetration retained by a projectile after passing through a target. A smaller value suggests that the target exerts significant resistance to the projectile’s penetration [35]. Both in the experimental and simulation results, there is a minimum value of DOP in the F-NC structure, indicating that the target plate is effective in hindering the projectile. The difference between the DOP obtained from the finite element simulation and the experimental measurement is within 8.0%, as shown in Table 5. Figure 8 illustrates the overall damage of the target plates after penetration. It can be observed that the F-NC target plate exhibits the least damage after penetration, while the I-C target plate shows cracking at the ceramic ball layer in addition to radial and secondary cracks, resulting in a lifting damage pattern. These results indicate that the numerical simulation exhibits similar failure mechanisms to the experimental. By comparing the simulated and experimental data, we observed a significant level of consistency, thereby validating the accuracy of the material parameters and models.

#### 3.3.2. Numerical Results

**Figure 9:**
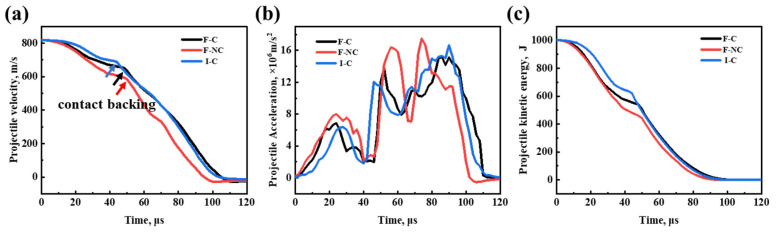
Histories of the projectile during penetration. (**a**) Velocity–time curve, (**b**) Acceleration–time curve, (**c**) Kinetic energy–time curve.

In addition to the macroscopic damage to the target plate shown in Figure 8, the variations in projectile velocity and kinetic energy can also directly reflect the penetration resistance of the target plate. As the bullet penetrates, it first encounters the B_4_C/Al composite material. Compared with the ceramic balls, the B_4_C/Al composite material is not effective in eroding the projectile, allowing the bullet to pass through at a high velocity. From Figure 9a,c, it can be observed that the F-NC target plate performs exceptionally well in reducing projectile velocity and kinetic energy. The time required for the projectile to pass through the F-NC target plate is longer, at 50 μs, compared to 46 μs for the F-C target plate and 42 μs for the I-C target plate, showing a significant improvement. The acceleration–time curve also reveals that the F-NC target plate exhibits the highest acceleration of the projectile, with the widest peak width, demonstrating superior deceleration effects on the projectile. During the penetration of the I-C target plate, the occurrence of the first peak in projectile acceleration is delayed, indicating that the ceramic balls play a primary role in both erosion and deceleration of the projectile compared to the composite material [11,28].

## 4. Discussion

### 4.1. Different Array Layouts

It is obvious that ceramic ball array layouts will greatly affect the penetration resistance from the results shown in Table 4 and Figure 7. Compared to the target plate with compact ceramic balls, the relatively softer composite material in the gaps between the ceramic balls helps alleviate stress concentration during penetration, thereby avoiding extensive damage caused by direct rigid contact between the ceramic balls. Such a buffer effect was also reported in Hu and Jiang’s research [24,28]. Simultaneously, the composite material alters the crack propagation path from “ceramic ball to ceramic ball” to “ceramic ball to composite to ceramic ball”, promoting crack nucleation and growth at the interface between the ceramic balls and composite material. This enhances the energy absorption capacity of the target plate.

**Figure 10:**
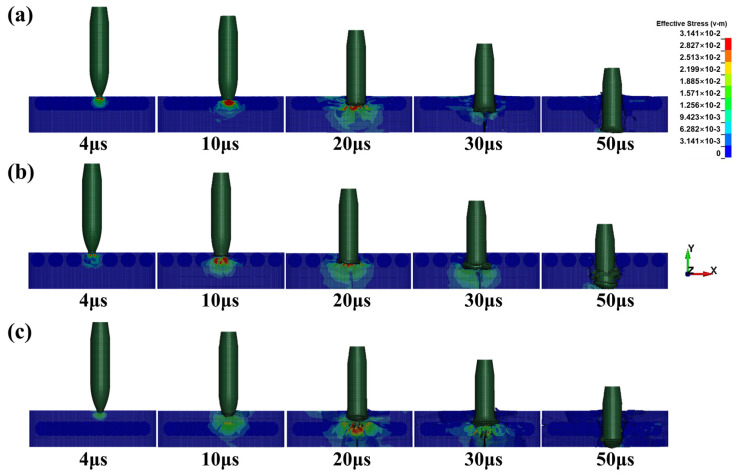
Stress distribution plots of three targets. (**a**) F-C structure, (**b**) F-NC structure, (**c**) I-C structure.

Numerical simulation results corroborate this point well. From the stress distribution maps of the target plates shown in Figure 10, it can be observed that the maximum stress occurs within the ceramic balls when the projectile contacts them, which aligns with expectations. As penetration continues, ceramic balls in contact with the projectile fail first due to stress transmission. In the F-C target plate, due to the compact arrangement of ceramic balls, stress easily propagates to the adjacent ceramic balls, leading to their failure, as shown in Figure 10a at 20 μs. Similar results are also confirmed in the I-C target plate. In contrast, the relatively softer composite material between the ceramic balls in the F-NC target plate acts as a buffer, reducing stress transmission. This prevents premature failure due to the hard-to-hard contact between the ceramic balls. Ensuring the integrity of the ceramic balls effectively prolongs the interaction time between the target plate and the projectile, allowing the anti-penetration capability of the ceramic balls to be fully utilized [17].

### 4.2. Support Effect by Composite

In addition to the role of constraint and dispersing stress, we find that the support provided by the composite material is also crucial in improving penetration resistance by comparing the F-C and I-C structures. According to the stiffness theory of laminated materials [36,37,38], when the ceramic balls are positioned inside, the supported composite material thickness is only half of the other two types, and its stiffness is only 1/8. When the bullet contacts the ceramic balls, composite material fails to provide sufficient support, resulting in extensive collapse. Additionally, it was also found that the target plate was partially lifted along the ceramic ball layer in the I-C target plate after penetration, performing the poorest penetration resistance among the three types of target plates.

A comparison of the damage observed in the F-C and I-C target plates in Figure 10 reveals that thinner support layers experience multiple crack failures during the repeated propagation of stress waves, as shown at 20 and 30 μs. Failure of the rear support leads to insufficient erosion of the projectile by the ceramic balls. Furthermore, when stress is transferred from the ceramic layer to the edges of the target plate, the plate exhibits lifting damage, resulting in overall failure.

In fact, both the constraint and support effects of the composite are designed to fully exploit the anti-penetration resistance of the ceramic. The projectile erosion by the ceramic occupies a major role in the ballistic performance [11,28].

### 4.3. Synergistic Effects between Ceramic Balls and Composite

Previous studies have shown that increasing the areal density of the target plate is beneficial for enhancing penetration resistance. However, among the three target plates considered in this study, the F-C target plate exhibits superior penetration resistance compared to the I-C target plate under the same areal density. Additionally, the F-NC target plate with lower areal density performs exceptionally well in terms of both target plate damage and DOP. Therefore, it is necessary to explore the impact of ceramic ball arrangement on the target plate’s penetration resistance from other aspects. Fortunately, the stress distribution and energy variation during the penetration process appear to provide solid evidence in this regard. The stress distribution has been well discussed in Figure 10.

**Figure 11:**
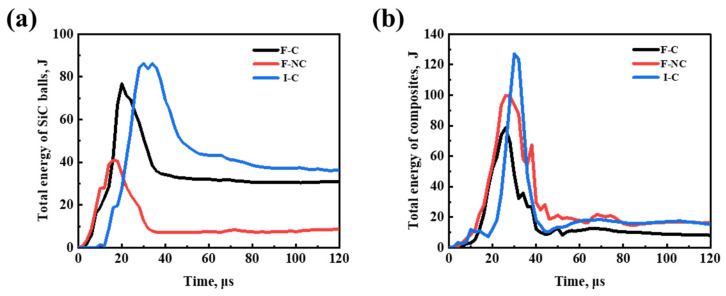
Total energy variations in the penetration process. (**a**) SiC balls, (**b**) B_4_C_p_/7075Al composite.

The energy–time profiles of SiC ceramic balls and composite materials shown in Figure 11 also provide strong evidence supporting the aforementioned viewpoint regarding the energy dissipation by the composite material buffer. The F-NC target plate, which exhibits the least energy dissipation by the ceramic balls, shows the smallest overall damage and superior penetration resistance. Overall, the target plate can demonstrate excellent penetration resistance only when the composite material effectively absorbs energy and minimizes ceramic ball failure. References [11,24] also clarify that ceramics primarily act as projectile erosion and composites act as energy absorbers.

In addition to projectile erosion, the initiation and propagation of cracks within the target plate are also important mechanisms for absorbing projectile kinetic energy, representing crucial research aspects in the protection field. Ceramic balls in the target plate promote crack nucleation and growth at the ball–composite interface, enhancing energy absorption. The expansion of cracks holds different implications for the target plate’s resistance against single and multiple penetrations. For single penetration, a greater number of crack initiation and deflection indicate increased absorption of projectile kinetic energy, thus improving the target plate’s penetration resistance. Conversely, maintaining the integrity of the target plate is more critical for multiple penetrations.

Due to the high strength of ceramic balls, some cracks tend to deflect around the ceramic balls, forming bridge cracks in the composite material, as shown in Figure 7(a1,a2). Such crack deflection not only enhances local energy absorption efficiency but also preserves the integrity of the target plate to the maximum extent, exhibiting excellent resistance against both single and multiple penetrations. Building upon this principle, the non-compact arrangement of ceramic balls, utilizing composite materials with lower modulus as buffers, serves to disperse stress and increase resistance to crack propagation from ceramic balls to composites to ceramic balls, further improving the energy absorption efficiency of the target plate [24,25].

Finally, we must state that there are some limitations in this paper, and we will continue to investigate the effect of the shape, size, and spacing of the individual ceramic units on the resistance to penetration in future studies. In addition, the difference in the penetration resistance at different locations due to the structural inhomogeneity of the composite armor is also one of the research directions.

## 5. Conclusions

To address the issue of overall failure in traditional ceramic panels during penetration, we developed a composite armor using independent ceramic ball units as the panel layer constrained by high-strength B_4_C/Al composite material. Using three different array arrangements, we investigated the respective roles and synergistic effects of the ceramic balls and composite material during the penetration process. These findings provide innovative insights for the design and fabrication of future composite armor based on independent ceramic units. The main conclusions are as follows:

1. The composite material with three different structures was prepared using the pressure infiltration method. The B_4_C particles and SiC ceramic balls are uniformly distributed within the composite material and tightly bonded to the metal matrix without noticeable defects such as voids. Surface undulations of the ceramic balls provide favorable conditions for mechanical interlocking between the composite material and the ceramic balls.
2. The F-NC structure exhibits the least macroscopic damage and DOP in the ballistic test, demonstrating the most outstanding penetration resistance performance. In contrast, the I-C structure experiences extensive spalling during penetration, indicating the poorest penetration resistance.
3. During penetration, the ceramic balls primarily contribute to projectile erosion and deceleration, while the composite material mainly serves the purpose of energy absorption and support. In the F-NC structure, the target plate has a longer interaction time with the projectile, highlighting the erosion and deceleration effects. This is attributed to the buffer effect provided by the relatively soft composite material in the gaps between the ceramic balls. It reduces stress transmission between the balls, mitigates ceramic ball damage, and enhances the penetration resistance capability of the target plate.

## Figures and Tables

**Table 1:** The details of the ceramic composite armor samples.

	F-C	F-NC	I-C
Density of B_4_C_p_/7075Al composite (g/cm^3^)	2.65	2.65	2.66
Relative density of B_4_C_p_/7075Al composite (%)	99.4	99.4	99.8
Area density (kg/m^2^)	55.7	54.0	55.6
Thickness (mm)	20.7	20.5	20.8

**Table 2:** The J–C model constants of the projectile and B_4_C/Al composite (unit: cm-g-μs) [9].

Material	ρ	g	pr	a	b	n	c	m	tm
projectile	7.85	0.77	0.29	0.014	0.0015	0.12	0	1	1768
B_4_C/Al composite	2.65	0.783	0.24	0.009	0.0025	0.11	0.072	2.34	933
	d1	d2	d3	d4	d5				
projectile	1.0	0	0	0	0				
B_4_C/Al composite	0.0261	0.263	0	0	8.4				

**Table 3:** The JH-2 model constants of SiC (unit: cm-g-μs) [11].

Material	ρ	g	a	b	c	m	n	t	sfmax
SiC	3.16	1.83	0.96	0.35	0.65	0.0045	1	0.0037	0.13
	hel	phel	beta	d1	d2	k1	k2	k3	fs
	0.13	0.059	1.0	0.48	0.48	2.047	0	0	1.2

**Table 4:** Ballistic test data for different composite armors.

	F-C	F-NC	I-C
Bullet speed (m/s)	827	825	821
DOP (mm)	1.00	0.65	1.52
*D*_1_ (cm)	5.77	2.64	7.68
*D*_2_ (cm)	2.12	3.87	4.89
*D* (cm)	4.37	3.31	6.44

**Table 5:** Validation of numerical simulation by the remaining depth of penetration (DOP).

	F-C	F-NC	I-C
DOP by experiment (mm)	1.00	0.65	1.52
DOP by simulation (mm)	1.08	0.64	1.64
Tolerance	+8.0%	−1.5%	+7.9%

## Data Availability

The data presented in this study are available on request from the corresponding author.

## Dual-use Research Statement

We have made the following statements to our paper titled “A Novel Multi-Scale Ceramic-Based Array (SiCb+B4Cp)/7075Al as Promising Materials for Armor Structure”.

Ø Explanation of Potential Risks: Our paper examines the protective properties of armor materials. The research is limited to providing some theoretical and experimental support for the development of impact dynamics only and does not pose a threat to public health or national security.
Ø Evaluation of Benefits to the General Public: Our research is limited to the academic field, which is beneficial to the development of material science. There is no risk to the general public.
Ø Compliance with Laws: As an ethical responsibility, we strictly adhere to relevant national and international laws about dual-use research. And we have considered and adhered to these regulations in our paper.
